# Geographic clustering of travel-acquired infections in Ontario, Canada, 2008–2020

**DOI:** 10.1371/journal.pgph.0001608

**Published:** 2023-03-17

**Authors:** Vinyas Harish, Emmalin Buajitti, Holly Burrows, Joshua Posen, Isaac I. Bogoch, Antoine Corbeil, Jonathan B. Gubbay, Laura C. Rosella, Shaun K. Morris

**Affiliations:** 1 MD/PhD Program, Temerty Faculty of Medicine, University of Toronto, Toronto, ON, Canada; 2 Dalla Lana School of Public Health, University of Toronto, Toronto, ON, Canada; 3 Epidemiology, Biostatistics and Occupational Health, McGill University, Montreal, QC, Canada; 4 Yale School of Public Health, Yale University, New Haven, CT, United States of America; 5 Division of Infectious Diseases, The Hospital for Sick Children, Toronto, ON, Canada; 6 Division of Infectious Diseases, Department of Medicine, Temerty Faculty of Medicine, University of Toronto, Toronto, ON, Canada; 7 Public Health Ontario, Toronto, ON, Canada; 8 Department of Laboratory Medicine and Pathobiology, Temerty Faculty of Medicine, University of Toronto, Toronto, ON, Canada; 9 Institute for Better Health, Trillium Health Partners, Mississauga, ON, Canada; 10 Department of Paediatrics, Temerty Faculty of Medicine, University of Toronto, Toronto, ON, Canada; 11 Child Health Evaluative Sciences, The Hospital for Sick Children, Toronto, ON, Canada; Federal University of Mato Grosso do Sul, BRAZIL

## Abstract

As the frequency of international travel increases, more individuals are at risk of travel-acquired infections (TAIs). In this ecological study of over 170,000 unique tests from Public Health Ontario’s laboratory, we reviewed all laboratory-reported cases of malaria, dengue, chikungunya, and enteric fever in Ontario, Canada between 2008–2020 to identify high-resolution geographical clusters for potential targeted pre-travel prevention. Smoothed standardized incidence ratios (SIRs) and 95% posterior credible intervals (CIs) were estimated using a spatial Bayesian hierarchical model. High- and low-incidence areas were described using data from the 2016 Census based on the home forward sortation area of patients testing positive. A second model was used to estimate the association between drivetime to the nearest travel clinic and incidence of TAI within high-incidence areas. There were 6,114 microbiologically confirmed TAIs across Ontario over the study period. There was spatial clustering of TAIs (Moran’s I = 0.59, p<0.0001). Compared to low-incidence areas, high-incidence areas had higher proportions of immigrants (p<0.0001), were lower income (p = 0.0027), had higher levels of university education (p<0.0001), and less knowledge of English/French languages (p<0.0001). In the high-incidence Greater Toronto Area (GTA), each minute increase in drive time to the closest travel clinic was associated with a 3% reduction in TAI incidence (95% CI 1–6%). While urban neighbourhoods in the GTA had the highest burden of TAIs, geographic proximity to a travel clinic in the GTA was not associated with an area-level incidence reduction in TAI. This suggests other barriers to seeking and adhering to pre-travel advice.

## Introduction

Prior to the COVID-19 pandemic, international travel was increasingly prevalent. In 2019, there were 1.5 billion international travellers, about half of whom travelled to low and lower middle-income countries [1, 2]. With increasing travel, more travellers are exposed to infectious agents not endemic to their departure region and more travel-acquired infections (TAI) occur[3, 4]. Pre-travel advice (PTA), which includes patient education on topics such as malaria prophylaxis, vaccination, food and water safety, and strategies for prevention of other vector-borne diseases, has been shown to decrease the rate of TAIs [5]. The World Health Organization recommends all travellers receive pre-travel advice, yet only 15–54% of travellers do so [4, 6–8]. Surveillance data indicates that upwards of 70% of travellers who become ill due to travel did not receive PTA [9, 10].

Up to 76% of travellers acquire a TAI [1, 11]. Most TAIs are mild and self-limited, such as traveller’s diarrhea. However, up to 8% of travellers become ill enough to seek clinical care during or after travel, and up to 1% of all travellers develop a febrile or systemic illness with elevated morbidity or mortality, such as malaria, enteric fever, dengue fever and chikungunya [9]. A quarter of all travellers report symptoms of TAI after completion of travel, signifying a significant burden of imported infections and healthcare needs [1]. During the 10-year period from 2009–2018, 4,947 imported cases of malaria and 1,536 imported cases of enteric fever were reported in Canada, about half of which were reported in Ontario [12, 13]. Among sub-national jurisdictions in North America, only New York State has a higher reported rate of malaria cases than Ontario [13, 14]. In addition to the morbidity caused by these TAIs, attributable medical costs in Ontario have been calculated to be CAD$4,558 per case of malaria and CAD$7,852 per case of enteric fever [15].

Previous studies have found that travellers visiting friends and relatives (VFR), business travellers, and those travelling with children, on short notice, or for extended durations are less likely to receive PTA [4, 16–19]. These same factors have been associated with increased incidence of TAI [9, 10, 20]. Quality of PTA also varies depending on the provider, with VFRs especially being less likely to consult a travel medicine specialist [6, 11, 19]. Thus, many of the highest incidence travellers are likely not receiving appropriate PTA, leading to missed opportunities for prevention of TAIs.

Ontario’s public health system is subdivided into 34 local public health units. Demographic factors and incidence of TAIs vary significantly between health units, with three health units–Toronto, Peel, and Ottawa–reporting almost 80% of provincial malaria cases [12]. Due to the large population sizes covered by these health three units (one to three million each), it would be impractical to distribute TAI prevention efforts at the level of the public health unit. Geographical analysis by postal code for targeted interventions has yielded specific outcomes for non-communicable diseases [21, 22], but has not previously been described for TAIs in a Canadian context.

Our objective was to review all laboratory confirmed cases of four common TAIs (malaria, enteric fever, dengue fever, and chikungunya) in Ontario between 2008–2020 to identify high-resolution geographical clusters. Our secondary objective was to explore the association between geographic proximity to travel clinics and neighbourhood-level burden of TAIs. We hypothesized that there would be clustering of TAIs in urban centres and that proximity to travel clinics (measured by drive time) would be associated with reduced neighbourhood-level TAI burden.

## Methods

### Ethics statement

Approval from The Hospital for Sick Children Research Ethics Board (REB) was obtained for this study (REB #1000068880).

### Study design and setting

Our study setting is Ontario, Canada’s most populous province with a population over 15 million as of 2022 [23]. Ontario has a universal single-payer health system that provides free access to a wide range of services including laboratory testing to the vast majority of residents. We reviewed all tests for malaria, enteric fever (caused by Salmonella enterica serovar Typhi or Paratyphi), dengue, and chikungunya processed at Public Health Ontario’s laboratory between July 15, 2008 to December 31, 2020. Public Health Ontario is Ontario’s provincial public health reference laboratory and conducts confirmatory testing for the listed TAIs across the province. Since residents were eligible for testing without additional cost, we can provide population-level estimates of disease burden robust to detection bias.

### Data source

PHO’s laboratory information system was queried for the time period of July 15, 2008 (date of implementation of the laboratory information system) to December 31, 2020. All test results for malaria (microscopy, rapid diagnostic tests [RDT], and polymerase chain reaction [PCR] tests), enteric fever (blood, bone marrow, urine, and stool cultures), dengue fever (IgM enzyme-linked immunosorbent assay [ELISA], IgG ELISA, and PCR tests), and chikungunya (IgM ELISA, IgG ELISA, and PCR tests) were extracted. Chikungunya coverage begins in 2015 while dengue coverage begins in 2008 with ELISA and PCR added in 2016. Each data point was collected at the test level and included the patient’s home forward sortation area (FSA) from their 6-digit postal code in addition to the specimen type, test performed, and test result. If the FSA was unavailable, the submitter’s FSA was provided instead. Each test was also assigned a unique patient identifier based on health card number, first name, last name, and date of birth.

### Outcome definition

The primary outcome of this study was the population-standardized incidence ratio of TAIs at the FSA level. The FSA is defined by the first three alphanumeric characters within a Canadian postal code and is a common level of geographic analysis. Each FSA comprises roughly 20,000 people but can vary in coverage due to heterogeneity in geography and population size (e.g., FSAs in northern Ontario are very large in area as the region is sparsely populated).

To determine an accurate count of unique TAI episodes, we identified a time period for attribution of positive tests to the same infection episode (as opposed to a reinfection episode). Repeat positive results outside this period were considered to represent a reinfection. For malaria, the defined period for persistently positive microscopy was set at 14 days, and at 90 days for RDT and PCR. The period was set at 14 days for enteric fever culture, dengue PCR, and chikungunya PCR. For dengue or chikungunya IgM ELISA, the period was set at 365 days. Due to small counts and similar incidence profiles (i.e., same vector and similar geographic distribution), dengue and chikungunya cases were pooled during analysis as ‘arboviruses’. The main analyses are presented with all diseases pooled together as TAIs, but disaggregated analyses can be found in the S1 Text.

### Exposures and covariate selection

The primary aim of this study was to identify any geographic clustering in TAI burden within Ontario. The secondary aim was to explore the association between geographic proximity to travel clinics and FSA-level burden of TAIs. In Canada, self-described travel clinics provide heterogeneous services from healthcare providers whose expertise in travel medicine varies considerably. To identify and include in our analysis only travel clinics that meet a minimum standard, we defined a travel clinic as a healthcare site designated by the Public Health Agency of Canada as a yellow fever vaccination centre [24]. All listed sites in Ontario were identified and geocoded with latitude and longitude coordinates using the Google Maps Geocoding application programming interface (API) [25]. Drive times were calculated from the centroid of each FSA polygon to each travel clinic using the Open Source Routing Machine API which determines the shortest path between a series of points on road networks [26]. This list of drive times was then filtered to obtain the drive time from each FSA to the closest travel clinic as a continuous variable in minutes.

Sociodemographic covariates of interest were defined *a priori* based on expert knowledge and obtained from Statistics Canada’s 2016 Census (https://www12.statcan.gc.ca/census-recensement/2016/dp-pd/index-eng.cfm) at the FSA level. Specifically, we included log-transformed median household income after tax as a continuous variable and immigration status, knowledge of official languages (English and French), ethnicity (Caribbean, African, Latin American, Middle Eastern, East and Southeast Asian, and South Asian), and education level (postsecondary certificate/diploma/degree) as proportions.

### Statistical analyses

A detailed and accessible overview of spatial modelling with Bayesian hierarchical models can be found elsewhere [27]. Counts of unique TAIs were used to calculate annual period-wide FSA-level case counts as well as population-standardized incidence ratios (SIRs). The expected number of cases was determined by multiplying the Ontario-wide rate of pooled cases by the population of each FSA. Smoothed SIRs and 95% posterior credible intervals (CIs) were estimated with a Besag spatial Bayesian hierarchical model (BHM), which accounts for statistical instability and uncertainty in small area incidence and are widely used for small-area rate analyses [28, 29]. Due to overdispersion of case counts and large number of FSAs with zero cases, we used a zero-inflated negative binomial base model. The Moran’s I test was used to test for global spatial autocorrelation in BHM-smoothed SIRs (i.e., if the spatial distribution of values can be explained by random chance) [30].

Posterior CIs were used to identify high- and low- incidence areas of TAIs, which were described using sociodemographic data from the 2016 Canadian census. High-incidence areas were defined as those with smoothed SIR 95% CIs greater than 1 (i.e., lower credible limit > 1), and low-incidence areas with smoothed SIR 95% CIs less than 1 (i.e., upper credible limit < 1). Moderate-incidence areas are those with smoothed SIR 95% CIs that cross 1. To test for significant differences in Census characteristics between groups, we used the Kruskall-Wallis and Wilcoxon rank sum tests. All statistical tests were two-sided and a p-value of <0.05 was considered significant. A second BHM was used to estimate the association between drive time to the nearest travel clinic and incidence of TAI within high-incidence areas, adjusted for potential confounders. We estimated the percent variance explained by our covariates by subtracting the variance of the posterior SIRs in the unadjusted model from the variance of the adjusted model, dividing the difference by the unadjusted variance, and then multiplying the quotient by 100 [31]. All analyses were done in the R programming language (R version 4.0.1, RStudio version 1.3.9, Boston, MA, USA).

## Results

Between July 15, 2008 and December 31, 2020, a total of 171,500 tests for malaria, enteric fever, dengue, and chikungunya were performed on 107,106 unique individuals. To avoid inappropriate clustering around health facilities, including those that serve large immigrant populations, we excluded 388 tests because of missing or invalid (i.e., outside of Ontario) patient home FSA. Of the 171,112 tests that were eligible, a total of 11,398 were positive for at least one of the four TAIs under study. Of these positive tests, 5,284 were excluded because they represented persistently positive repeat test results for the same TAI episode in the same patient. Therefore, the final analytic cohort included 6,114 laboratory confirmed unique TAI episodes (Fig A in S1 Text).

**Fig 1:**
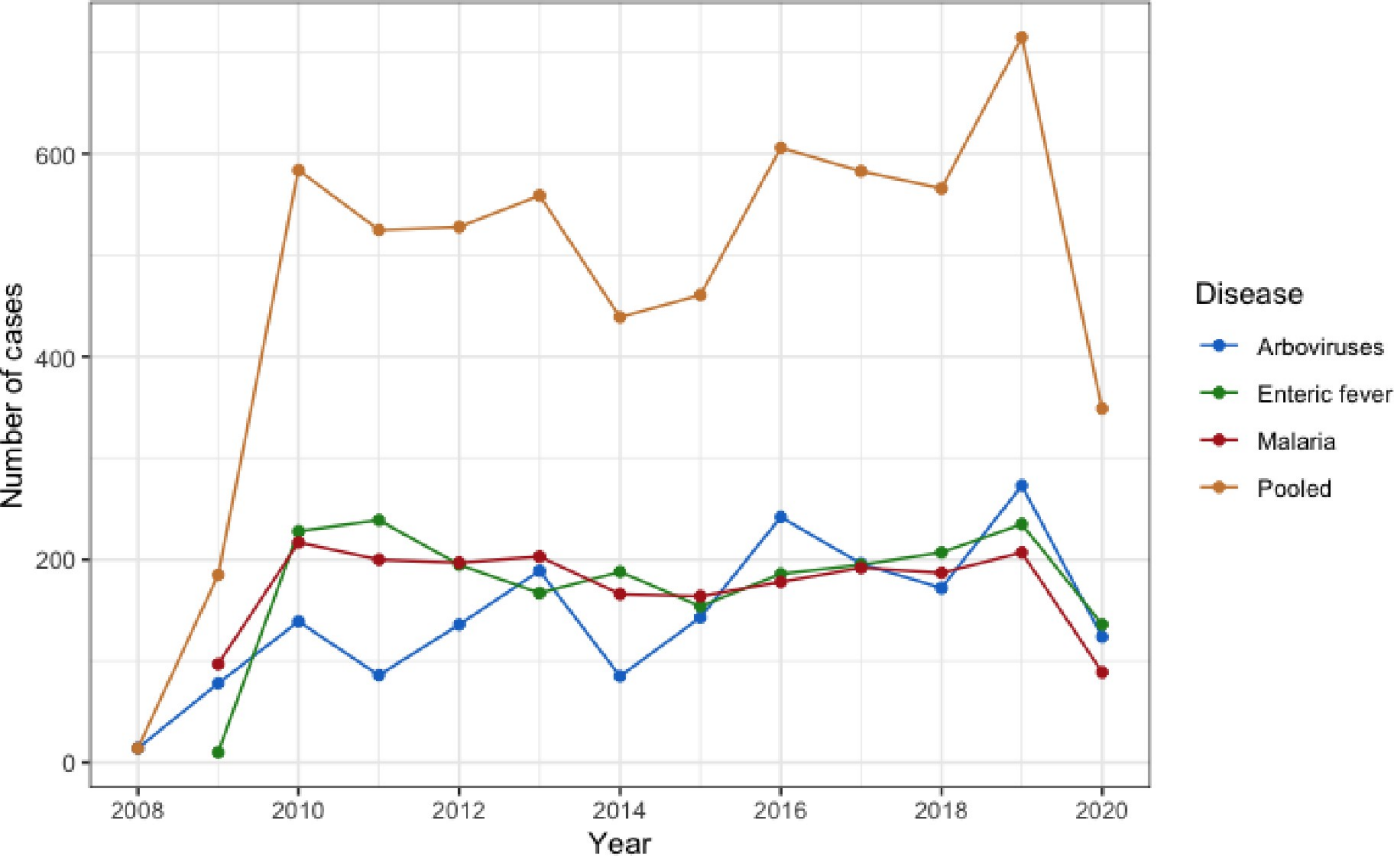
Year-over-year burden of travel-acquired infections in Ontario, Canada. The arbovirus category consists of both dengue and chikungunya.

The annual absolute number of microbiologically confirmed enteric fever and malaria infections remained relatively stable at around 200 per year between 2010 and 2019 (Fig 1). There was greater variation in arbovirus infection numbers over the same time period, with annual numbers fluctuating between 100 and 280. Pooled annual TAI episodes peaked in 2019 at 715. The incidence of all TAIs dropped sharply in 2020, coinciding with the COVID-19 pandemic.

**Fig 2:**
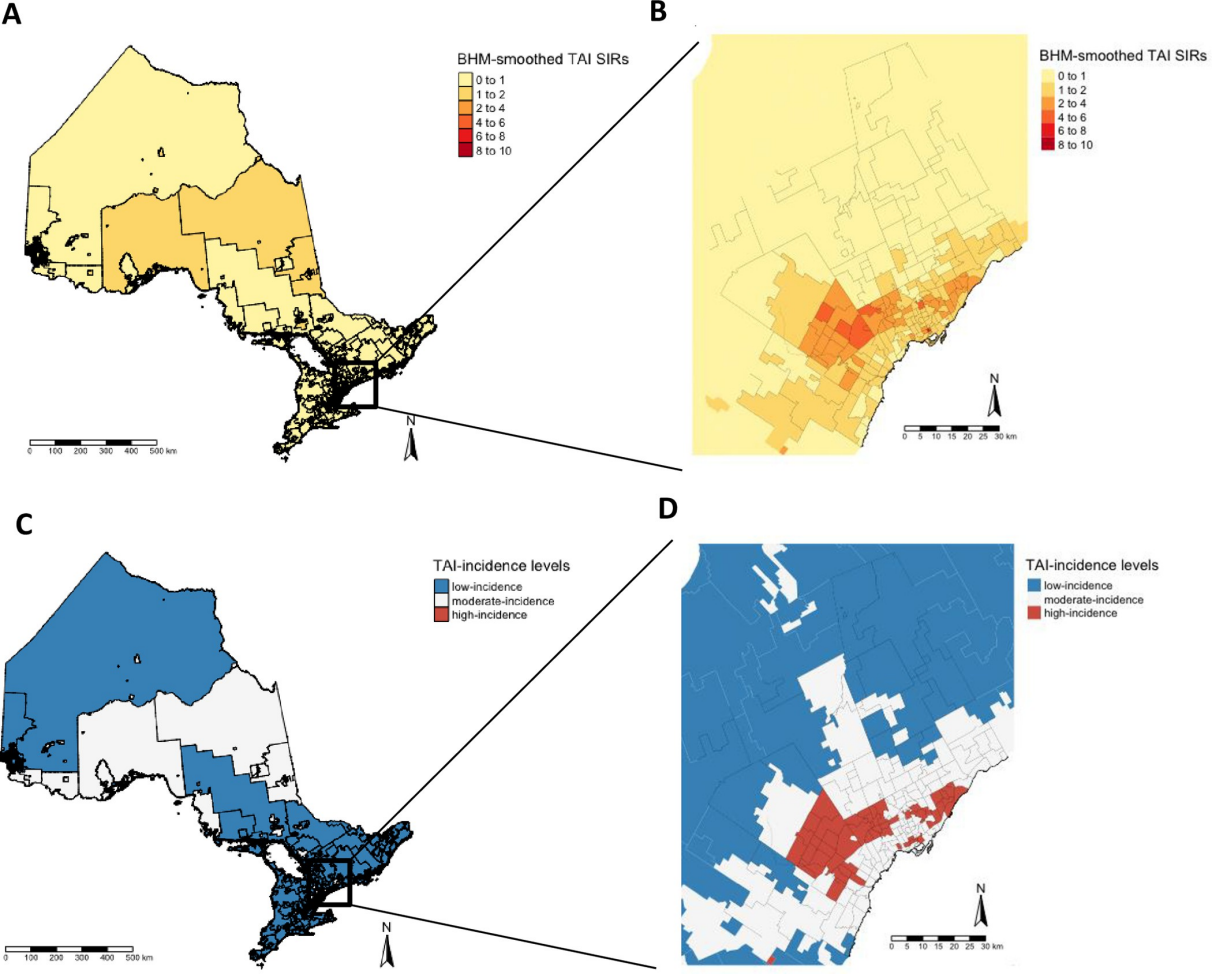
Standardized travel-acquired infection incidence across Ontario, Canada. Bayesian hierarchical model (BHM) smoothed standardized incidence ratios (SIRs) for travel-acquired infections (TAIs) and estimated incidence levels (panels A and C) with insets for the Greater Toronto Area (B and D). High-incidence areas are defined as those with smoothed SIR 95% credible intervals (Cis) greater than 1 and low-incidence areas with smoothed SIR 95% CIs less than 1. Adapted from Statistics Canada, 2016 Census–Boundary Files, 2019-11-13. This does not constitute an endorsement by Statistics Canada of this product.

BHM-smoothed TAI SIRs by FSA ranged from 0 to 8 across Ontario, with higher SIRs generally located in the Greater Toronto Area (GTA) (Fig 2A and 2B). There was spatial clustering of TAIs (Moran’s I = 0.59, p<0.0001). The majority of FSAs deemed high-incidence for TAIs (i.e., those with SIR 95% CIs greater than 1; n = 57) were located in the GTA (n = 53), with the majority of the remaining GTA FSAs considered moderate-incidence (Fig 2D). There were five FSAs with SIRs over 5, and three of those 5 corresponded to the locations of hospitals within downtown Toronto and Ottawa. The majority of FSAs outside the GTA had SIRs less than 1, with the exception of certain FSAs in Northern Ontario and the Greater Ottawa Area (Fig 2C and 2D).

**Fig 3:**
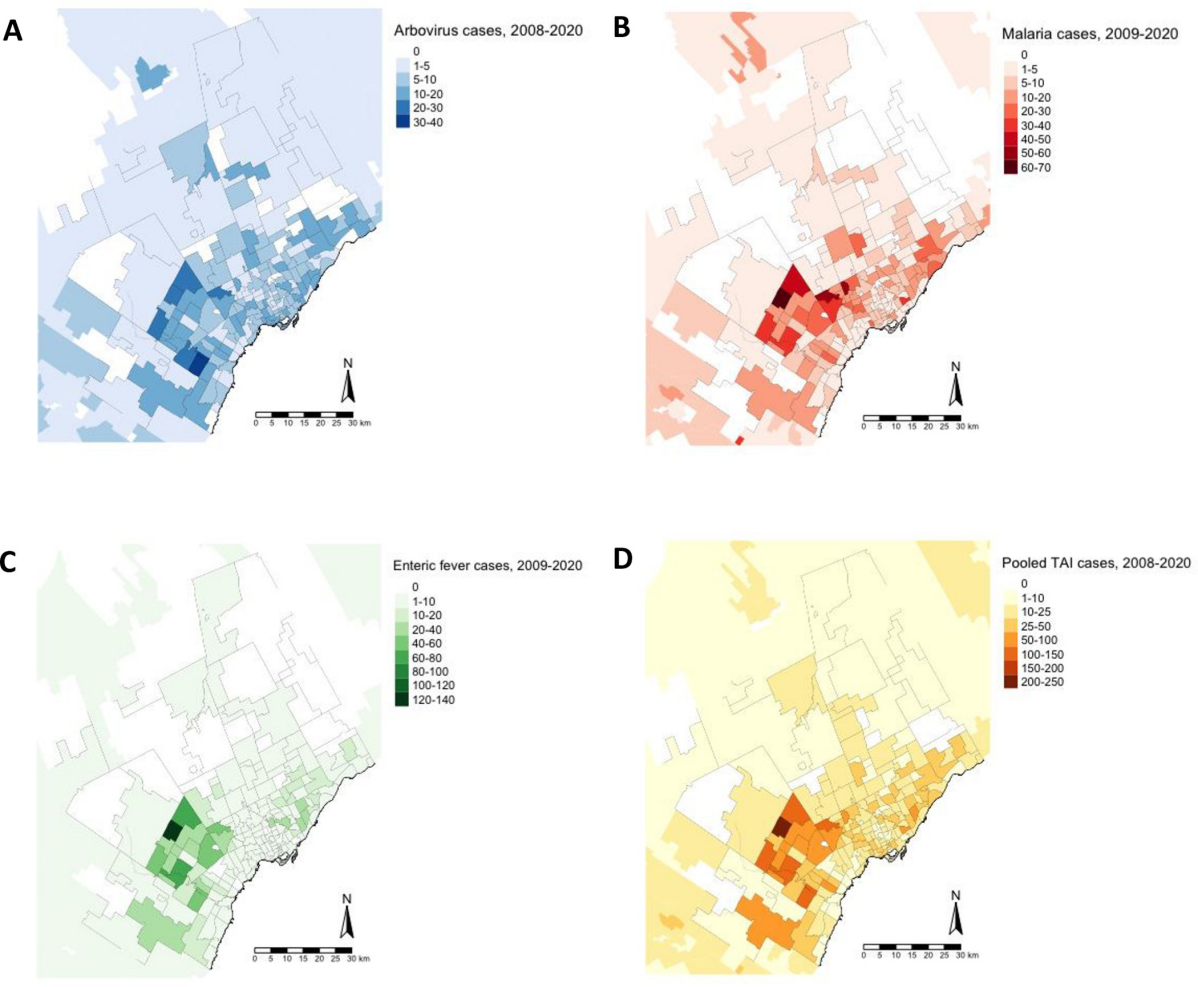
Burden of travel-acquired infections in the Greater Toronto Area. Raw counts of microbiologically confirmed infections for arboviruses (panel A), malaria (panel B), enteric fever (panel C) and for all diseases (panel D) in the Greater Toronto Area over the study period. Adapted from Statistics Canada, 2016 Census–Boundary Files, 2019-11-13. This does not constitute an endorsement by Statistics Canada of this product.

**Fig 4:**
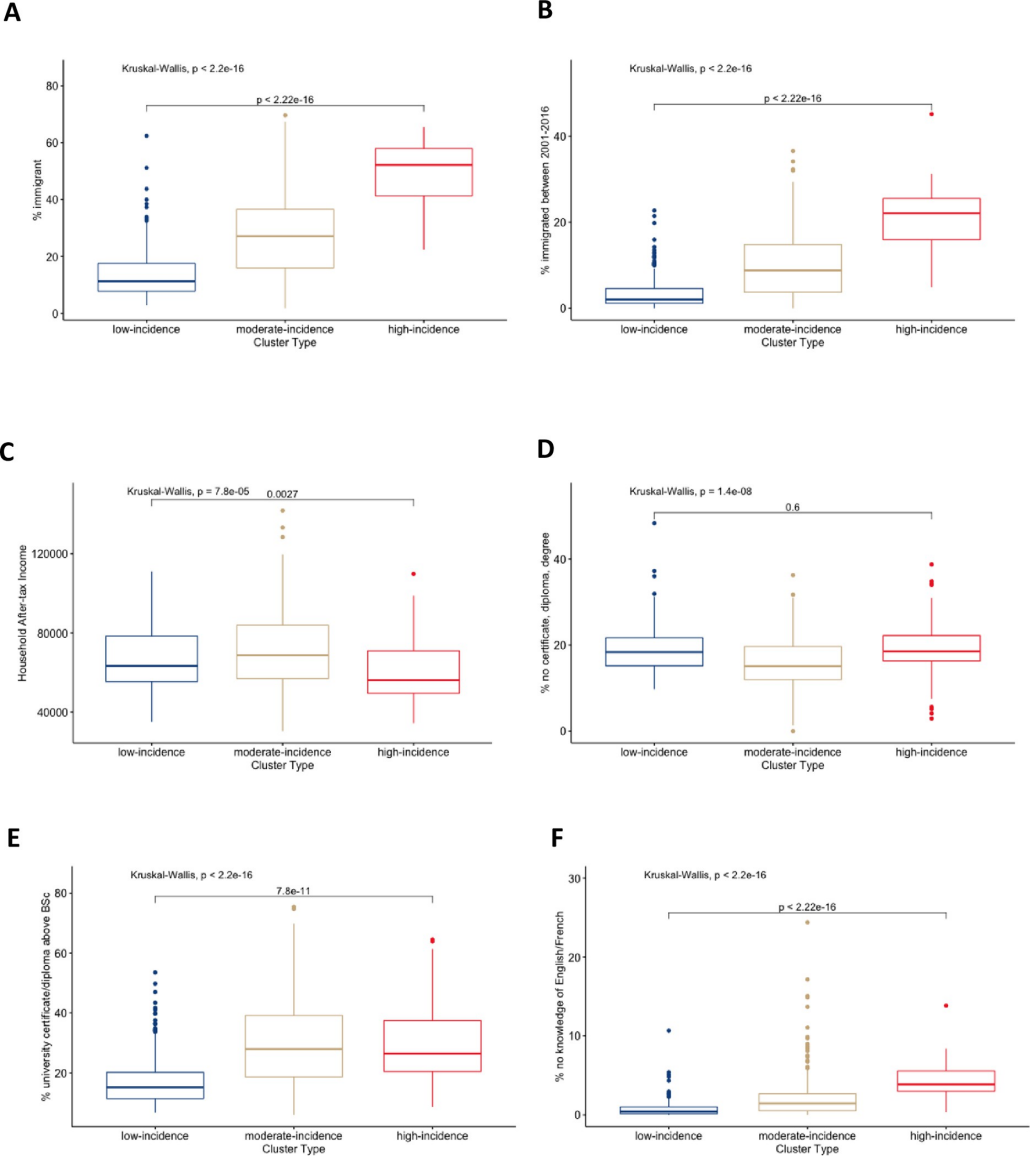
Characterizing high, moderate, and low-incidence clusters of travel-acquired illness burden across Ontario, Canada. Boxplots comparing clusters of FSAs across Ontario using key characteristics from the 2016 Census. Kruskall-Wallis and Wilcoxon rank sum tests were used as appropriate. All statistical tests were two-sided and a p-value of <0.05 was considered significant.

The absolute case counts in the GTA for all three disease categories—arboviruses, enteric fever, and malaria—generally followed the spatial pattern observed for the TAI SIRs (Fig 3). Enteric fever cases were the most clustered (Fig 3C) while arboviruses were the most dispersed (Fig 3A). Higher incidence FSAs had a significantly higher proportion of: immigrants; recent immigrants (migrated between 2001–2016); lower household after-tax income; university certificate or diploma above a Bachelors; and lower knowledge of Canada’s official languages English and French (Fig 4). In the GTA, each minute increase in drive time to the closest travel clinic was associated with a 3% reduction in TAI incidence (95% CI 1–6%) (Table 1). When comparing adjusted and unadjusted models, the Census covariates explained roughly 15% of the variation in TAI incidence.

**Table 1:** Drivetime analysis. Adjusted Bayesian hierarchical model estimates for the association between drivetime to closest travel clinic and forward sortation area (FSA)-level travel-acquired infection burden within the Greater Toronto Area.

FSA-level Incidence Factor	Adjusted Relative Incidence (95% CI)
Drivetime to closest travel clinic	0.966 (0.943–0.988)
% of population with no knowledge of English/French	1.014 (0.963–1.069)
% of population that is immigrant	0.980 (0.961–1.000)
% of population with Caribbean ethnicity	1.042 (1.004–1.082)
% of population with African ethnicity	1.034 (0.990–1.079)
% of population with Latin American ethnicity	1.013 (0.949–1.081)
% of population with Middle Eastern ethnicity	1.039 (1.004–1.074)
% of population with East/Southeast Asian ethnicity	1.009 (0.994–1.024)
% of population with South Asian ethnicity	1.031 (1.019–1.044)
% of population with postsecondary certificate/diploma/degree	0.998 (0.979–1.017)
Log-transformed median household after-tax income	0.888 (0.543–1.513)

## Discussion

We reviewed all laboratory confirmed cases of four common TAIs—malaria, enteric fever, dengue fever, and chikungunya—in Ontario between 2008–2020 to identify high-resolution geographical clusters. There was spatial clustering of TAIs largely within the GTA, the largest urban centre in Canada. Compared to low-incidence areas, high-incidence areas had higher proportions of immigrants, lower income status, higher university education, and lower knowledge of English and French. Contrary to our hypothesis, each minute increase in drivetime to the closest travel clinic in the GTA was associated with a 3% reduction in TAI incidence.

Previous work in Ontario has suggested that area-level measures of socioeconomic status are not fully representative as proxies for individual-level data, but that they can measure important drivers of health outcomes and inequities [32]. This is particularly important in the context of TAIs. Communities of immigrants may live within similar regions and may share comparable incidences of TAI because of similar travel destinations and/or behaviours associated with travel. We found that Census covariates explained only 15% of the TAI incidence variation between adjusted and unadjusted models, thus other community- and individual-level factors are likely at play. Ethnicity was an important lens for understanding TAI clusters and provides important potential pathways for targeted education. In the GTA, enteric fever was highly clustered in predominantly South Asian communities whereas malaria was clustered in predominantly African communities (particularly Nigerian and Somali communities). These findings correlate with the high incidence rates of enteric fever in South Asia and of malaria in sub-Saharan Africa, likely due to community members recently immigrating from or travelling to these regions. This clustering of TAIs based on travel region may explain the significant spatial autocorrelation in TAIs observed here (Moran’s I = 0.59, p<0.0001), which is higher than some of the other correlations reported in the literature such as heat-related illness (Moran’s I = 0.21) and opioid-related deaths (Moran’s I = 0.46) [33, 34]. The highest incidence neighbourhoods in this study are marginalized along multiple social determinants of health including low economic status, recent immigration, and lower knowledge of Canada’s official languages. Intersectional vulnerability in communities has been linked to a range of adverse health outcomes, from chronic conditions to COVID-19 [35, 36]. While it may appear counterintuitive that higher levels of university education were associated with TAI clusters, this could be because many immigrants were highly educated in their home countries prior to their arrival in Canada and that education was favoured in the Canadian points-based immigration system until the early 2010s [37]. However, this finding may be limited to our jurisdiction and we caution readers against making a similar argument in their jurisdiction if circumstances and evidence may differ. Geospatial analyses may create meaningful lines of inquiry, including by challenging conventional knowledge, that are well suited for further exploration by mixed-methods and qualitative research in high-incidence areas.

Access to health services has been conceptualized along three dimensions: physical accessibility, financial affordability, and acceptability [38]. Regarding physical accessibility, our drive time analysis demonstrates that clinics appear to be appropriately located near high-incidence communities. Financial affordability has been previously reported to be a barrier to seeking and following PTA among VFR travellers residing in the GTA [39]. Similar to the United States, Ontario’s universal health insurance program does not cover PTA, travel-related immunizations nor chemoprophylaxis [40]. Given this barrier and the potentially high healthcare costs linked to the management of TAIs, our findings highlight the need for cost effectiveness analyses evaluating the financial benefits of travel clinics on TAI prevention as well as pilot programs for PTA cost coverage in high-incidence communities [15]. The last dimension of access to health services, acceptability, may be the most complex barrier to address as it encompasses numerous dimensions of risk perception [41]. Studies have found that travellers may not accurately perceive risks associated with their travel and thus may believe PTA to be unnecessary for them [7, 19]. These beliefs are accentuated in VFR travellers. Uncertainty regarding itineraries and rushed circumstances surrounding travel (e.g., to attend a funeral) provide logistical challenges that hinder the feasibility of seeking PTA [39]. Since the tools to prevent these TAIs are different and vary in effectiveness, geospatial analyses, both aggregated and disaggregated by pathogen of interest, may serve as the first step towards guiding the local co-design of interventions with trusted community organizations to promote the acceptability of PTA in high-incidence communities [16, 39, 42, 43]. There can be significant ethno-cultural heterogeneity within a high-incidence community and thus clinicians and public health professionals may face the need to provide several distinct cultural tailorings of geographically targeted interventions (i.e., malaria prevention tailored to Nigerian residents, typhoid prevention tailored to South Asian residents). Research into cost-effective and culturally sensitive interventions to improve uptake of PTA could represent a fruitful area for future work.

Our study features numerous strengths and contributions. First, it leverages a comprehensive dataset from a universal single-payer health system. Since residents were eligible for testing without additional cost, we can provide population-level estimates of disease burden robust to detection bias. Moreover, Ontario has a combination of highly urban and rural communities and is among the most ethnically diverse populations in the world, both of which improve the generalizability of our work to high income jurisdictions with high levels of immigration and multicultural populations. Spatial analyses may be especially useful to plan interventions in major metropolitan areas, as similar ethno-cultural clustering of TAIs has been reported in VFRs living in New York City and London [44–47]. Large, representative studies of this patient population and timescale are currently rare, as many of the published studies are based on chart reviews from individual travel clinics, surveys of travellers in airports, or the International Society of Travel Medicine GeoSentinel Surveillance Network collaboration efforts [9, 16]. While GeoSentinel provides a wide-ranging survey of the burden of TAIs across countries, the relatively fewer number of participant sites per country prevents comprehensive studies within a given geospatial jurisdiction. To our knowledge, we are the first group to examine geographic access to travel clinics as a form of pre-event access prior to a health outcome of interest (TAI). Finally, our geospatial approach was rigorous as we used Bayesian modelling to smooth small case counts and computed drive times to measure distance to care as opposed to less accurate straight-line distances. Bayesian modelling also reduces bias due to the modifiable areal unit problem (MAUP), whereby aggregating data into geopolitical units to study trends is sensitive to the administrative boundaries creating these units [48].

Our study also has limitations. Although we expect to have excellent case identification, a limitation is that those without coverage in Ontario’s health insurance plan (e.g., newly landed immigrants/refugees) may be less likely to seek care and be identified with a TAI. Moreover, we did not have patient-level information on the location, nature, and duration of travel, as well as if PTA was individually sought or received. Lacking these details limits our ability to fully understand patterns and incidence groups. Our analysis does not consider differences in travel clinics (e.g., if a certified travel medicine specialist provides care, clinics that provide pre-travel advice without yellow fever certification) and changes in travel clinic locations over time. Unfortunately, no historical repository of travel clinic locations is publicly available. Our analysis also does not consider changes in community demographics over time since we only used data from the 2016 Census to gauge risk factors. As with all area-level studies, it is possible that our findings would be different based on different geographic boundaries due to the MAUP [49]. While smaller geographic boundaries may capture more variation, larger ones may be more relevant for planning interventions [50], reduce the risk of spillover effects [51], and are likely more temporally stable. Future work in Ontario should leverage mixed-methods designs to glean more nuanced, individual level information in the identified high-incidence communities. Jurisdictions with year-specific risk factors could also consider more advanced methods that handle time-varying confounding (e.g., G methods) to account for changing trends. Moreover, there may be misclassification bias due to the difficulty in determining if a positive test result corresponds to a new clinical episode or persistent infection. To mitigate this, we set time cut-offs between positive tests based on the expected persistence of a positive test result, acknowledging that this approach may still yield misclassified episodes. Finally, while PHO performs the majority of testing for TAIs in Ontario, it is possible that some individuals may have obtained testing by other laboratory facilities and would not be captured in this study. As PHO only performs bacterial stool cultures to support outbreaks or at the request of public health units, it is possible that our estimates undercount enteric fever as diagnosed by stool. Since arboviral infections are more likely to be mild and self-limited compared to malaria and enteric fever, it is possible our estimates undercount the burden of dengue and chikungunya.

## Conclusion

While urban neighbourhoods in the Greater Toronto Area had the highest burden of travel-acquired infections in Ontario, geographic proximity to a travel clinic was not associated with an area-level reduction in the incidence of infections. This suggests other barriers to seeking and/or adhering to pre-travel advice. Future research, policy measures, and community-based interventions should consider barriers to the affordability and acceptability of pre-travel advice to better understand and ultimately reduce the burden of travel-acquired infections.

## Supporting information

S1 Text (DOCX)
