# Supplementary material for: Geographic clustering of travel-acquired infections in Ontario, Canada, 2008–2020
Source: PLOS Glob Public Health. 2023 Mar 17;3(3):e0001608. doi: 10.1371/journal.pgph.0001608 (PMC10022755; doi:10.1371/journal.pgph.0001608)
Supplement: S1 Text — (DOCX) [file pgph.0001608.s001.docx]

**Geographic clustering of travel-acquired infections in Ontario, Canada, 2008-2020**

**Supplementary Materials**

**Vinyas Harish^1,2^, Emmalin Buajitti^2,3^, Holly Burrows^4^, Joshua Posen^5^, Isaac I. Bogoch^6^,**

**Antoine Corbeil^7^, Jonathan Gubbay^7,8^, Laura Rosella^2,8^, Shaun K. Morris^5,9,10 *^**

^1^ MD/PhD Program, Temerty Faculty of Medicine, University of Toronto, Toronto, ON, Canada

^2^ Dalla Lana School of Public Health, University of Toronto, Toronto, ON, Canada

^3^ Epidemiology, Biostatistics and Occupational Health, McGill University, Montreal, QC, Canada

^4^ Yale School of Public Health, Yale University, New Haven, CT, USA

^5^ Division of Infectious Diseases, The Hospital for Sick Children, Toronto, ON, Canada

^6^ Division of Infectious Diseases, Department of Medicine, Temerty Faculty of Medicine, University of Toronto, Toronto, ON, Canada

^7^ Public Health Ontario, Toronto, ON, Canada

^8^ Department of Laboratory Medicine and Pathobiology, Temerty Faculty of Medicine, University of Toronto, Toronto, ON, Canada

^9^ Department of Paediatrics, Temerty Faculty of Medicine, University of Toronto, Toronto, ON, Canada

^10^ Child Health Evaluative Sciences, The Hospital for Sick Children, Toronto, ON, Canada

* **Corresponding Author: Shaun K. Morris**

Division of Infectious Diseases, The Hospital for Sick Children

555 University Ave., Toronto, ON, M5G 1X8, Canada

[shaun.morris@sickkids.ca](mailto:shaun.morris@sickkids.ca)

**Contents:**

**Section 1- Outcome definitions:**

Table A – Time between test results for chikungunya Pg 3

Table B – Time between test results for dengue Pg 3

Table C – Time between test results for enteric fever Pg 4

Table D – Time between test results for malaria Pg 4

Fig A – Flowchart of test results Pg 5

**Section 2- Disaggregated analyses:**

Fig B – Ontario-wide maps for each disease in raw counts Pg 6

Fig C – Bayesian-hierarchical model standardized incidence ratios for arboviruses Pg 7

Fig D – Census analysis for arboviruses Pg 8

Fig E – Bayesian-hierarchical model standardized incidence ratios for enteric fever Pg 9

Fig F – Census analysis for enteric fever Pg 10

Fig G – Bayesian-hierarchical model standardized incidence ratios for malaria Pg 11

Fig H – Census analysis for malaria Pg 12

**Section 3- Other spatial analyses:**

Table E – Global Moran’s values Pg 13

Table F – Unadjusted drivetime analysis in the Greater-Toronto Area Pg 14

**Section 1- Outcome definitions:**

**Table A.** Distribution of repeat testing for chikungunya in patients who had multiple tests. Note these are all tests, not only positive tests.

| **Test type - PCR** | | |
| --- | --- | --- |
| ***Interval between first and last tests*** | ***Number of tests within interval*** | ***Number of tests outside interval*** |
| 14 days | 82 | 6 |
| 30 days | 86 | 2 |
| 60 days | 86 | 2 |
| 90 days | 86 | 2 |
| 180 days | 87 | 1 |
| 365 days | 87 | 1 |
| **Test type – IgM ELISA** | | |
| ***Interval between first and last tests*** | ***Number of tests within interval*** | ***Number of tests outside interval*** |
| 14 days | 305 | 253 |
| 30 days | 401 | 157 |
| 60 days | 458 | 100 |
| 90 days | 472 | 86 |
| 180 days | 496 | 62 |
| 365 days | 510 | 48 |

**Table B.** Distribution of repeat testing for dengue in patients who had multiple tests. Note these are all tests, not only positive tests.

| **Test type - PCR** | | |
| --- | --- | --- |
| ***Interval between first and last tests*** | ***Number of tests within interval*** | ***Number of tests outside interval*** |
| 14 days | 83 | 7 |
| 30 days | 86 | 4 |
| 60 days | 87 | 3 |
| 90 days | 88 | 2 |
| 180 days | 88 | 2 |
| 365 days | 88 | 2 |
| **Test type – IgM ELISA** | | |
| ***Interval between first and last tests*** | ***Number of tests within interval*** | ***Number of tests outside interval*** |
| 14 days | 960 | 787 |
| 30 days | 1199 | 548 |
| 60 days | 1345 | 402 |
| 90 days | 1399 | 348 |
| 180 days | 1464 | 283 |
| 365 days | 1520 | 227 |

**Table C.** Distribution of repeat testing for enteric fever in patients who had multiple tests. Note these are all tests, not only positive tests.

| **Test type - Culture** | | |
| --- | --- | --- |
| ***Interval between first and last tests*** | ***Number of tests within interval*** | ***Number of tests outside interval*** |
| 14 days | 5255 | 3614 |
| 30 days | 6208 | 2661 |
| 60 days | 7021 | 1848 |
| 90 days | 7346 | 1523 |
| 180 days | 7703 | 1166 |
| 365 days | 7999 | 870 |

**Table D.** Distribution of repeat testing for malaria in patients who had multiple tests. Note these are all tests, not only positive tests.

| **Test type – Microscopy** | | |
| --- | --- | --- |
| ***Interval between first and last tests*** | ***Number of tests within interval*** | ***Number of tests outside interval*** |
| 14 days | 3591 | 667 |
| 30 days | 3767 | 491 |
| 60 days | 3869 | 389 |
| 90 days | 3911 | 347 |
| 180 days | 3971 | 287 |
| 365 days | 4034 | 224 |
| **Test type – RDT / PCR** | | |
| ***Interval between first and last tests*** | ***Number of tests within interval*** | ***Number of tests outside interval*** |
| 14 days | 3823 | 655 |
| 30 days | 3995 | 483 |
| 60 days | 4095 | 383 |
| 90 days | 4136 | 342 |
| 180 days | 4195 | 283 |
| 365 days | 4258 | 220 |

171, 500 tests for pooled TAIs performed on 107,106 unique individuals between July 15^th^ 2008 and December 31^st^ 2020 obtained from PHO

Exclude 388 tests due to missing or invalid (i.e., outside of Ontario) patient home FSA

171,112 tests

Exclude 159, 714 negative tests

11,398 positive tests

Exclude 5,284 positive test results that likely represent non-unique TAI episodes

6,114 positive tests that likely correspond to unique TAI episodes

**Fig A.** Flowchart describing process of arriving at the final analytic cohort. TAI, travel-acquired infection; PHO, Public Health Ontario; FSA, forward-sortation area

**Section 2- Disaggregated analyses:**

**A**


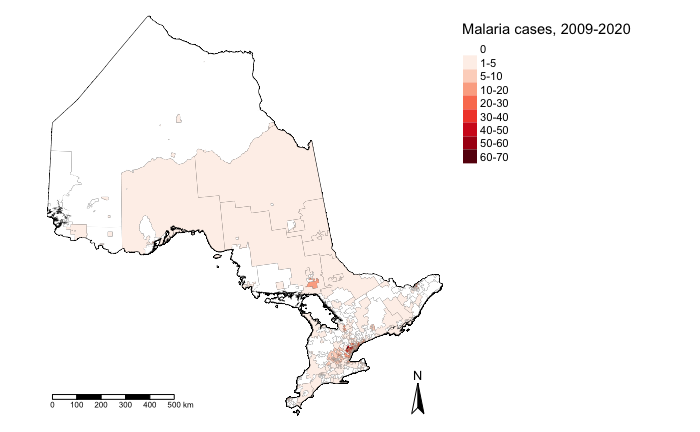


**B**


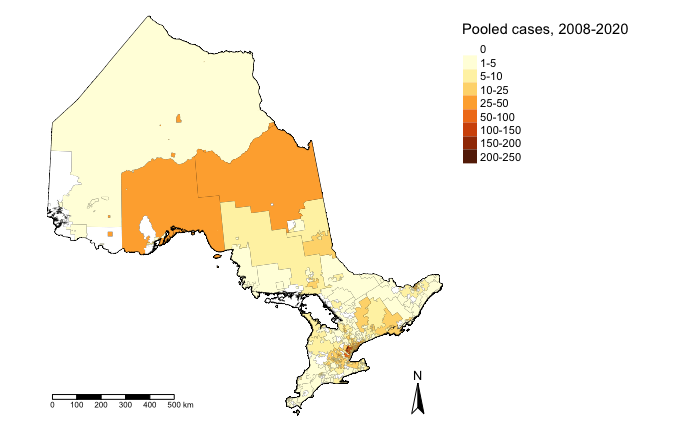

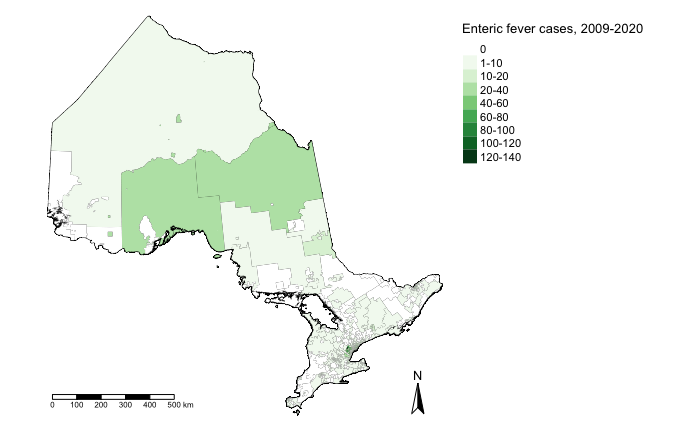

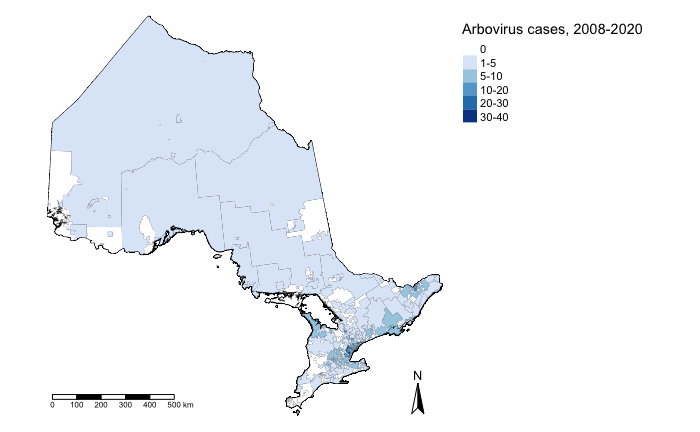


**D**

**C**

**Fig B.** Ontario-wide maps of travel-acquired infections. Adapted from Statistics Canada, 2016 Census – Boundary Files, 2019-11-13. This does not constitute an endorsement by Statistics Canada of this product.

**B**

**A**


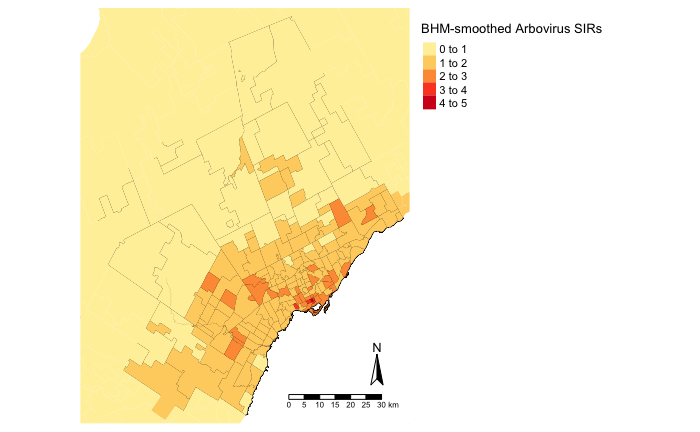

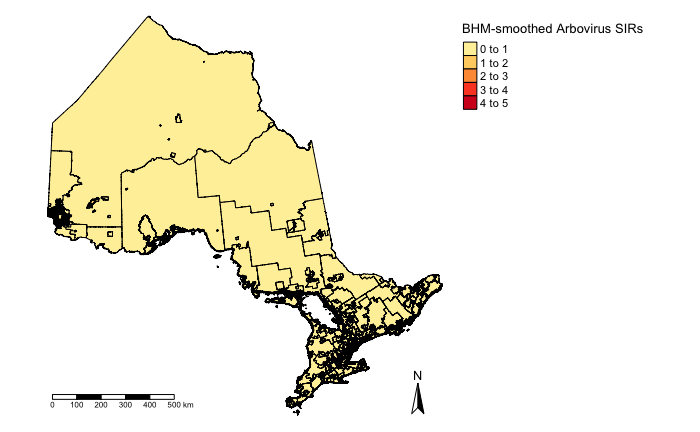


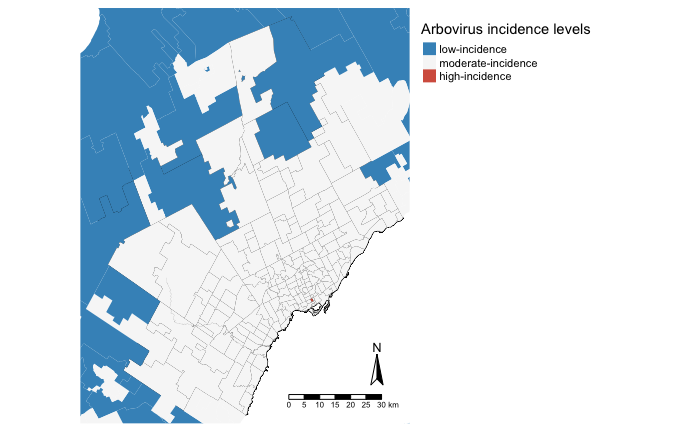


**D**

**C**


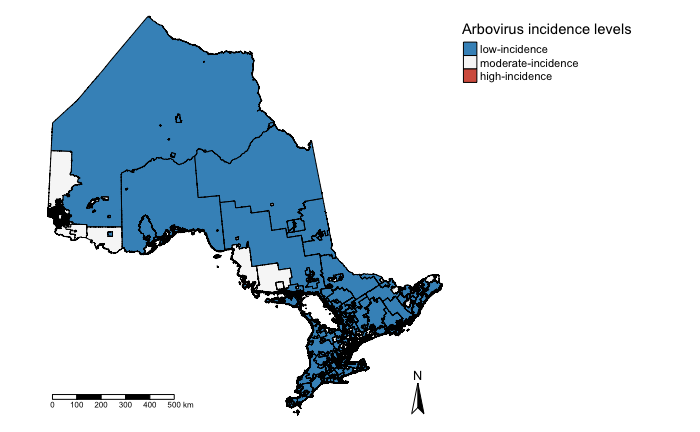


**Fig C.** Bayesian hierarchical model (BHM)-smoothed arbovirus standardized incidence ratios (SIRs, panels A, B) and incidence levels (C,D) Ontario-wide (A,C) and in the Greater Toronto Area (B,D). . Adapted from Statistics Canada, 2016 Census – Boundary Files, 2019-11-13. This does not constitute an endorsement by Statistics Canada of this product.


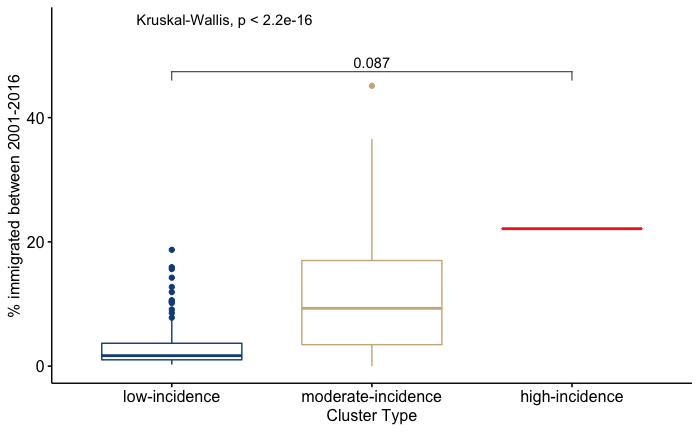

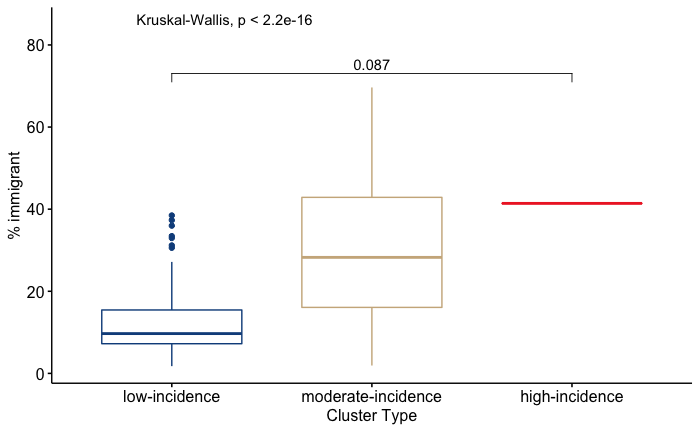


**D**

**C**

**B**

**A**


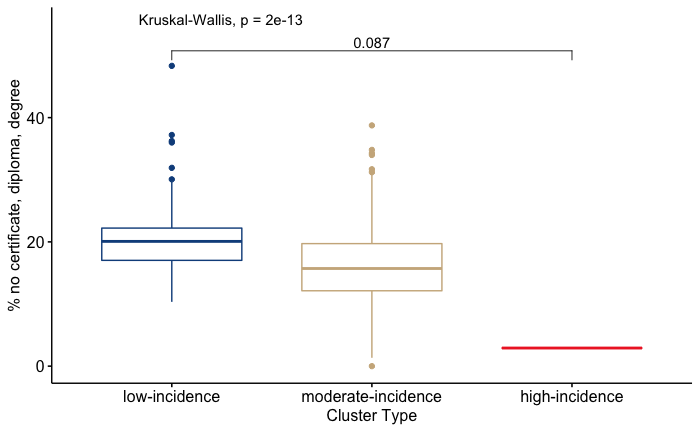


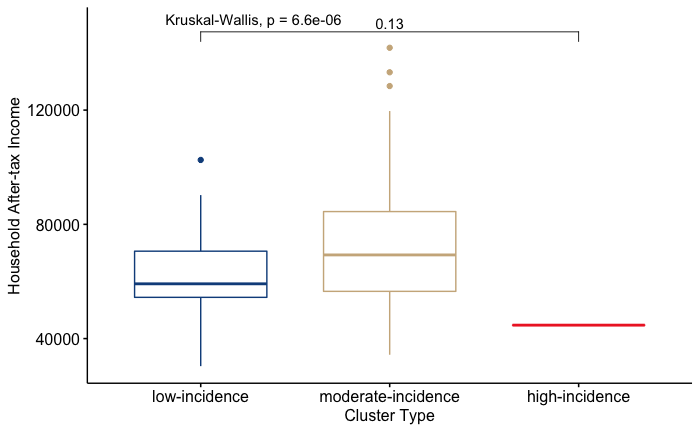


**E**

**F**


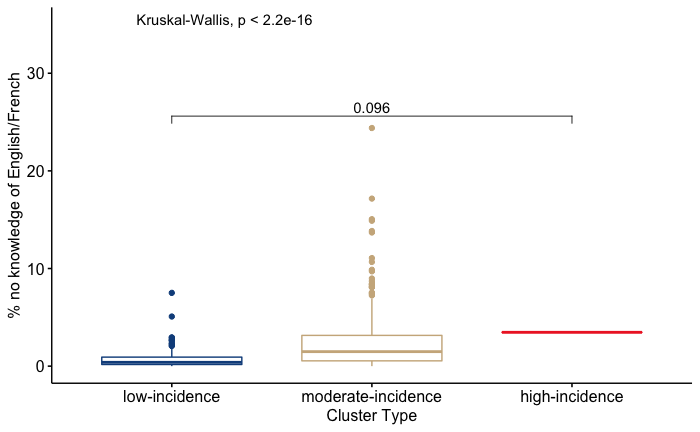


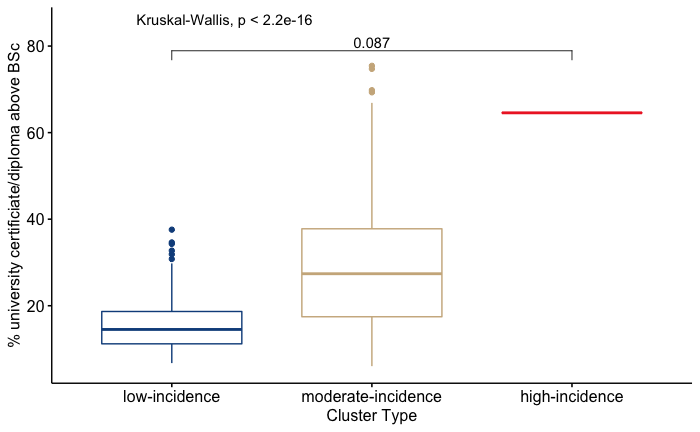


**Fig D.** Boxplots comparing high, moderate, and low arbovirus incidence clusters of FSAs across Ontario using key characteristics from the 2016 Census. Kruskall-Wallis and Wilcoxon rank sum tests were used as appropriate. All statistical tests were two-sided and a p-value of <0.05 was considered significant


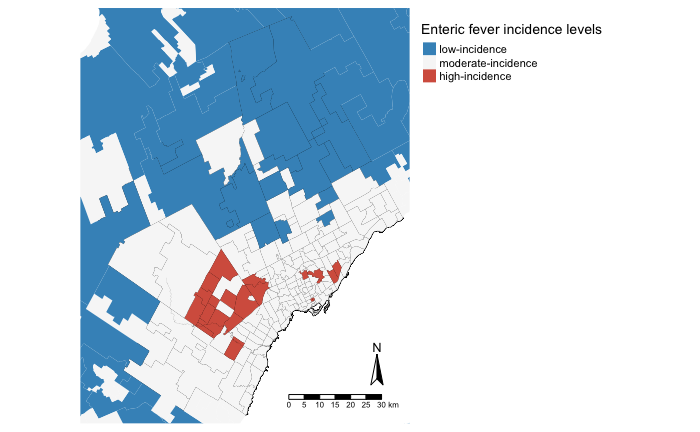

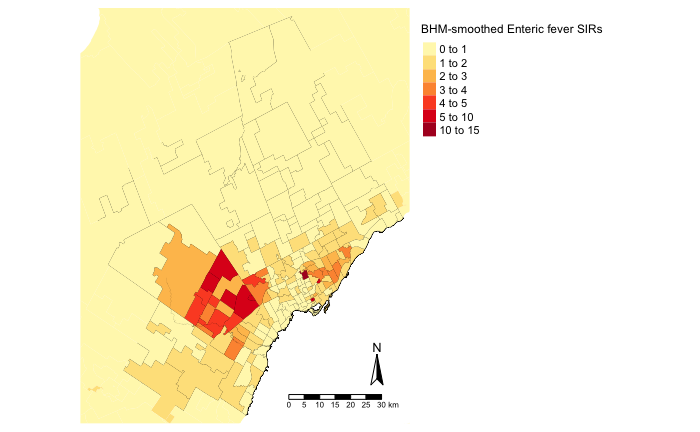

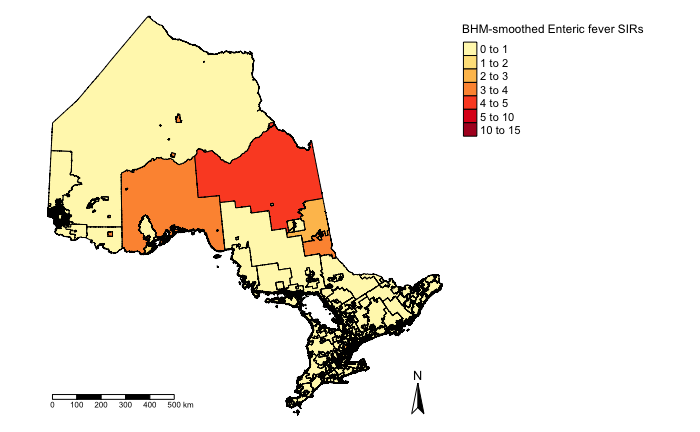


**B**

**A**

**D**

**C**


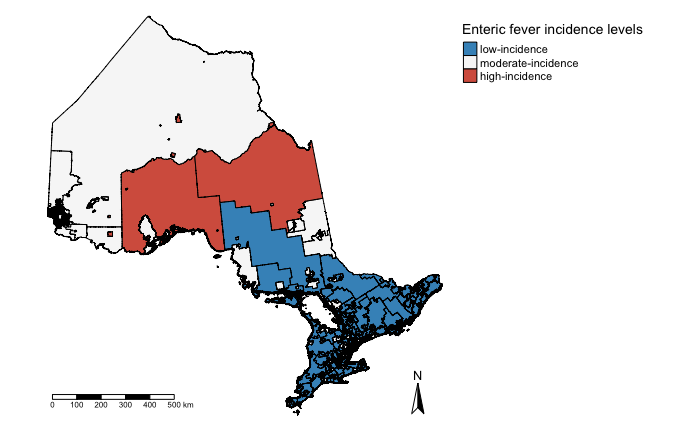


**Fig E.** Bayesian hierarchical model (BHM)-smoothed enteric fever standardized incidence ratios (SIRs, panels A, B) and incidence levels (C,D) Ontario-wide (A,C) and in the Greater Toronto Area (B,D). Adapted from Statistics Canada, 2016 Census – Boundary Files, 2019-11-13. This does not constitute an endorsement by Statistics Canada of this product.


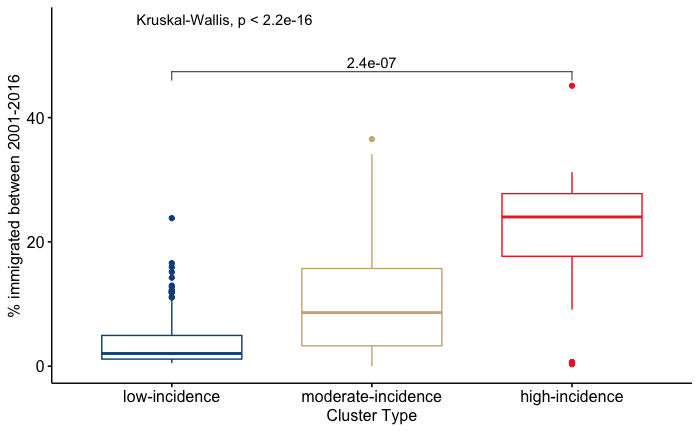

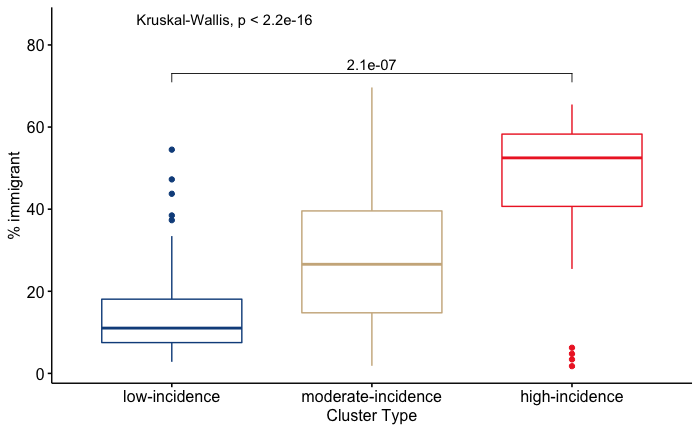


**D**

**C**

**B**

**A**


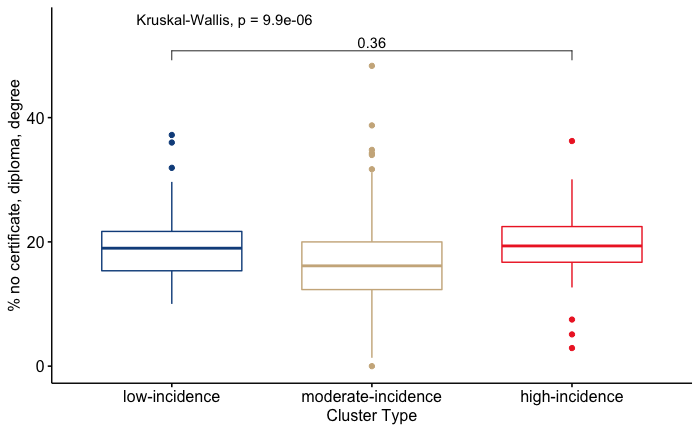


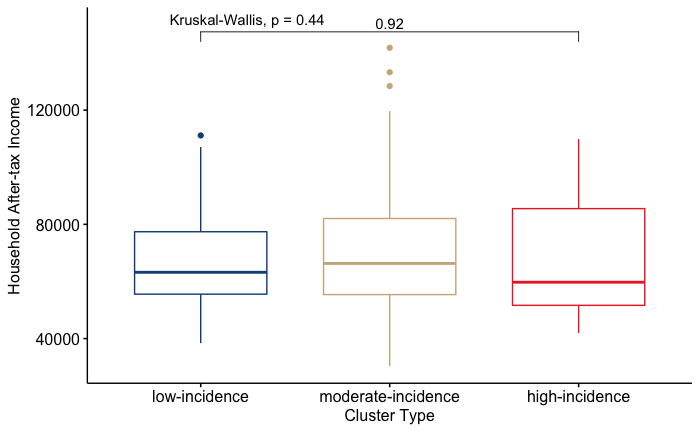


**E**

**F**


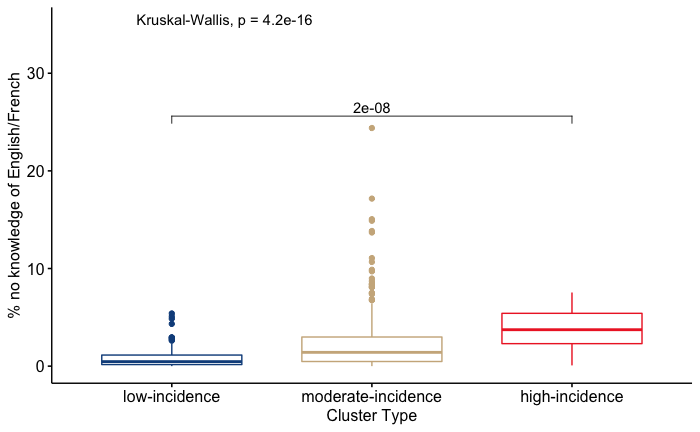

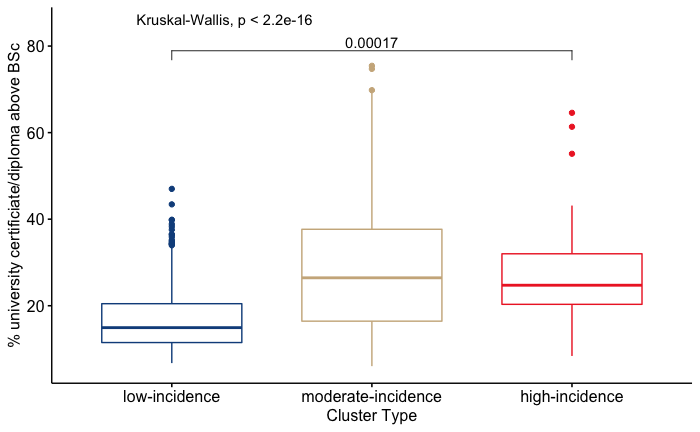


**Fig F.** Boxplots comparing high, moderate, and low enteric fever incidence clusters of FSAs across Ontario using key characteristics from the 2016 Census. Kruskall-Wallis and Wilcoxon rank sum tests were used as appropriate. All statistical tests were two-sided and a p-value of <0.05 was considered significant


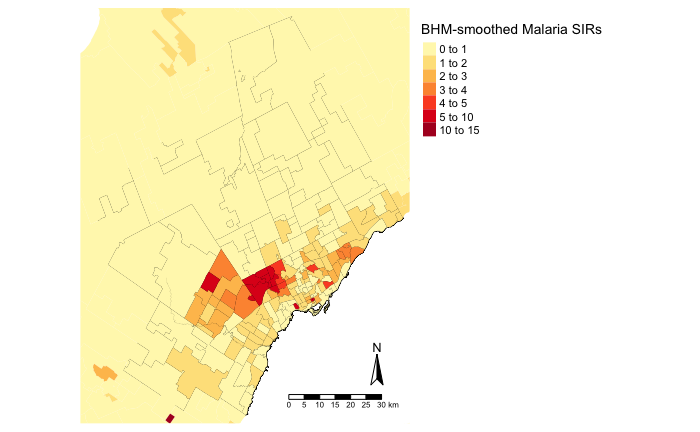

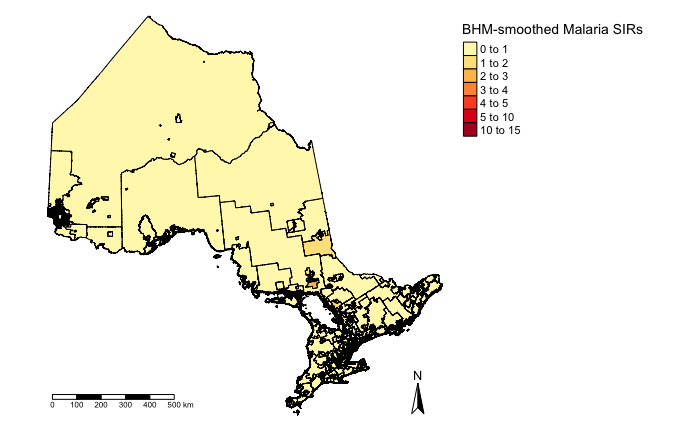


**B**

**A**


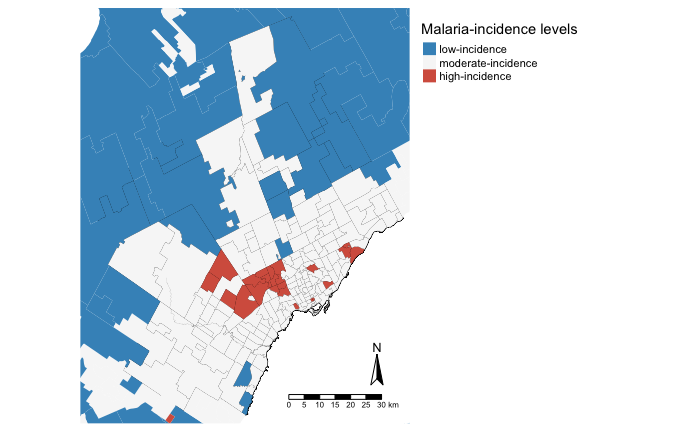


**D**

**C**


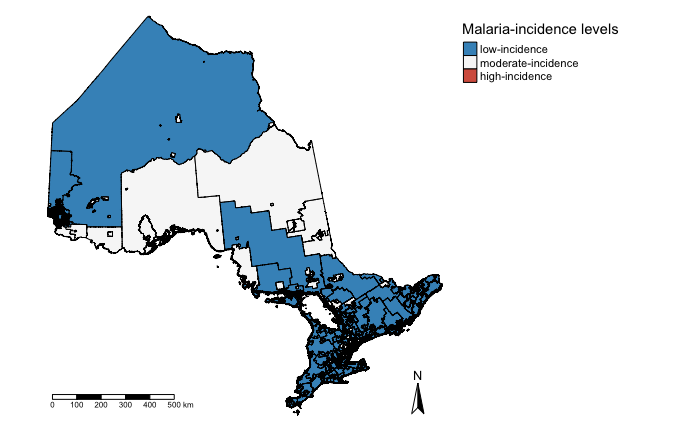


**Fig G.** Bayesian hierarchical model (BHM)-smoothed malaria standardized incidence ratios (SIRs, panels A, B) and incidence levels (C,D) Ontario-wide (A,C) and in the Greater Toronto Area (B,D). Adapted from Statistics Canada, 2016 Census – Boundary Files, 2019-11-13. This does not constitute an endorsement by Statistics Canada of this product.


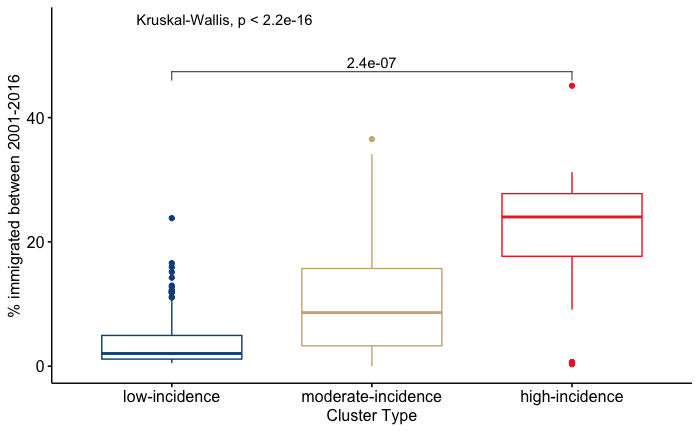

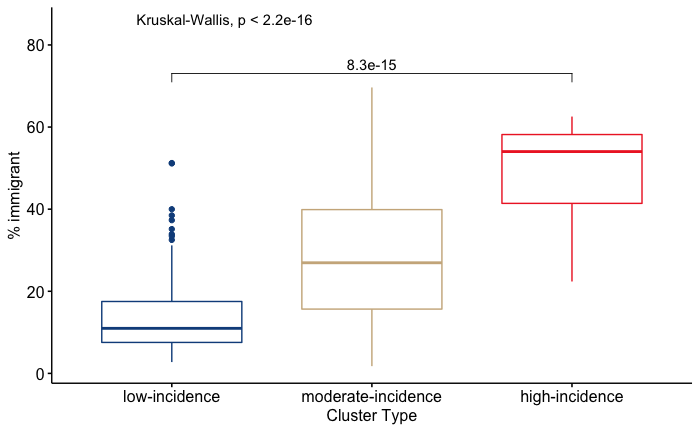


**B**

**A**


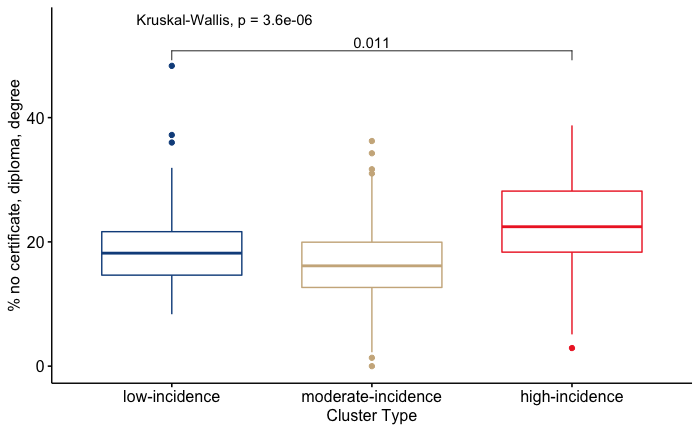


**D**

**C**


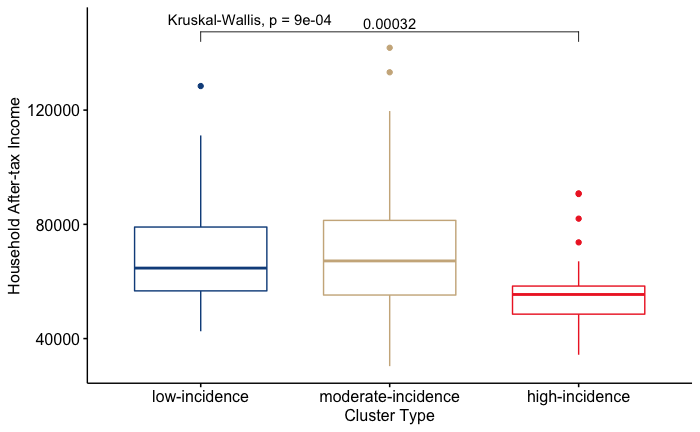


**E**

**F**


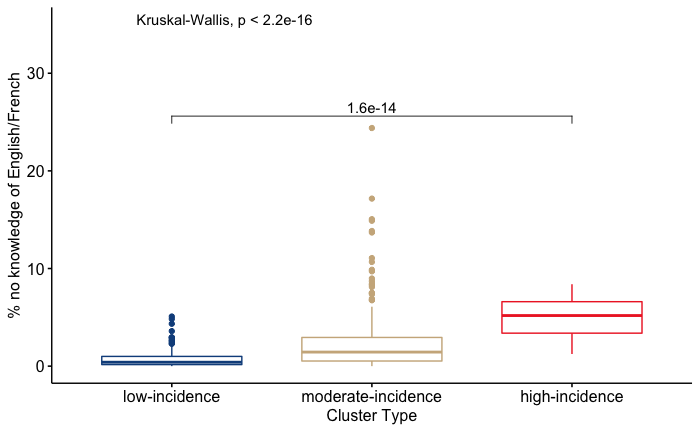


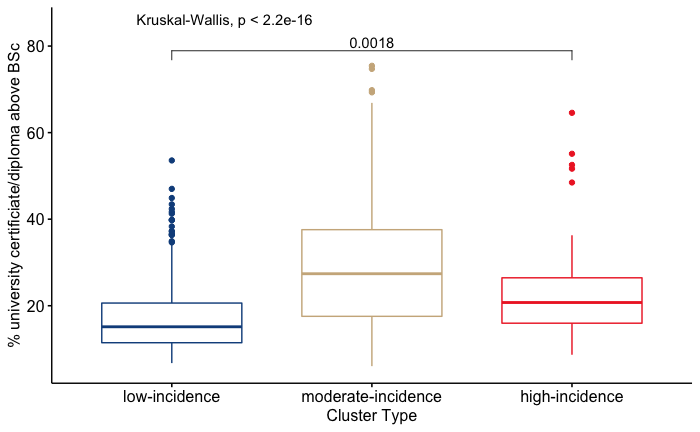


**Fig H.** Boxplots comparing high, moderate, and low malaria incidence clusters of FSAs across Ontario using key characteristics from the 2016 Census. Kruskall-Wallis and Wilcoxon rank sum tests were used as appropriate. All statistical tests were two-sided and a p-value of <0.05 was considered significant.

**Section 3- Other analyses:**

**Table E.** Global Moran’s values for the four travel-acquired infections in Ontario, both as raw numbers of cases and as Bayesian hierarchical model (BHM)-smoothed standardized incidence ratios (SIRs). A Global Moran’s statistic of -1 means the data are perfectly dispersed, 1 means fully clustered, and 0 means random. The null hypothesis is that the data are randomly dispersed and the alternate hypothesis is that the data are more spatially clustered than expected by chance alone.

| **Travel-acquired infection** | **Global Moran’s Value** | **P Value** |
| --- | --- | --- |
| Arboviruses (raw cases) | 0.4070292097 | < 2.2 x 10^-16^ |
| Arboviruses (BHM SIRs) | 0.8581881539 | < 2.2 x 10^-16^ |
| Enteric Fever (raw cases) | 0.3465345508 | < 2.2 x 10^-16^ |
| Enteric Fever (BHM SIRs) | 0.4648618627 | < 2.2 x 10^-16^ |
| Malaria (raw cases) | 0.3330814755 | < 2.2 x 10^-16^ |
| Malaria (BHM SIRs) | 0.454405101 | < 2.2 x 10^-16^ |
| Pooled (raw cases) | 0.3784245201 | < 2.2 x 10^-16^ |
| Pooled (BHM SIRs) | 0.5944372992 | < 2.2 x 10^-16^ |

**Table F.** Unadjusted Bayesian hierarchical model estimates for the association between drivetime to closest travel clinic and forward sortation area-level travel-acquired infection burden within the Greater Toronto Area.

| **FSA-level Risk Factor** | **Adjusted Relative Risk (95% CI)** |
| --- | --- |
| Drivetime to closest travel clinic | 0.9436499 (0.9175942 - 0.9694756) |
